# A series of N-of-1 trials to assess the therapeutic interchangeability of two enalapril formulations in the treatment of hypertension in Addis Ababa, Ethiopia: study protocol for a randomized controlled trial

**DOI:** 10.1186/s13063-017-2212-0

**Published:** 2017-10-10

**Authors:** Chalachew Alemayehu, Geoffrey Mitchell, Abraham Aseffa, Alexandra Clavarino, James McGree, Jane Nikles

**Affiliations:** 1 Faculty of Medicine University of Queensland, Brisbane, QLD Australia; 2 Armauer Hanson Research institute, Jimma Road, ALERT Compound, Addis Ababa, Ethiopia; 3 School of Pharmacy, University of Queensland, Brisbane, QLD Australia; 4 Queensland University of Technology, Brisbane, QLD Australia; 5 UQCCR, Brisbane, QLD Australia

**Keywords:** Hypertension, Enalapril, Therapeutic equivalence, N-of-1 trial, Randomized controlled trial, Generic

## Abstract

**Background:**

Hypertension is one of the leading causes of morbidity and mortality in Ethiopia. Treatment usually involves lifelong medication use. Enalapril is a common drug for the treatment of hypertension in Ethiopia. However, the drug is expensive and, therefore, there is limited capacity for people to afford the treatment. Locally produced Enalapril is a cost-effective solution to treat the disease. However, as local medicines regulation does not include bioequivalence tests on locally produced drugs, physicians and patients need assurance about the effectiveness and safety of local generics. Evidence on therapeutic equivalence is needed on these untested local drugs.

**Methods:**

This is a hospital-based, randomized, partially blinded, three-cycle crossover trial in single patients, comparing a locally produced version of enalapril with enalapril imported from Europe. Patients involved in this trial are not blinded, as there is no local facility to produce relatively small numbers of placebos or encapsulated drugs. To ensure blinding of study investigators and data analysts, study medications are prepared by an independent pharmacy unit using opaque medication packaging. The importance of maintaining blinding is also part of patient pre-trial education. Each N-of-1 trial will consist of three successive 14-day treatment pairs, each pair comprising 7 days of 5–20 mg local and 7 days of 5–20 mg imported enalapril taken once daily in the morning. The primary outcome will be the average difference in systolic blood pressure as measured by home blood pressure measurements.

**Discussion:**

The number of locally produced products, such as enalapril, being approved without proof of bioequivalence is dramatically increasing. By bridging the information gap on bioequivalence, the trial will give rigorous evidence on therapeutic equivalence of locally produced enalapril in the treatment of hypertension. If there is no difference, the hypothesized result, then patients can take the local medicine with confidence. This trial will also will determine whether aggregated N-of-1 studies are feasible to evaluate untested generic drugs in resource-limited countries where bioequivalence testing centers are unavailable.

**Trial registration number:**

Australian and New Zealand Clinical Trial Registry, ID: ACTRN12616001088437p. Registered on 12 August 2016.

**Electronic supplementary material:**

The online version of this article (doi:10.1186/s13063-017-2212-0) contains supplementary material, which is available to authorized users.

## Background

Ethiopia is the second most populous country in Africa with a population size of 100,936,943 [1]. In 2011, the World Health Organization (WHO) estimated that 34% of the Ethiopian population dies from non-communicable diseases, with a national cardiovascular disease prevalence of 15% [2]. In particular, hypertension is a major public health challenge because of its high frequency and associated risks. Ethiopian studies have found a significantly high prevalence of hypertension at the workplace (27.3%) [3] and in an urban community (30%) [4]. In 2008, the Ethiopian Federal Ministry of Health (FMOH) identified hypertension as the seventh leading cause of mortality [5]. This makes it the single most important cause of mortality from non-communicable diseases in Ethiopia.

Drug therapy for hypertension involves the use of a series of drug classes and often requires taking multiple drugs, which makes the treatment expensive for patients. In order to provide better access to affordable drugs, the Ethiopian Government legalized the production and commercialization of locally produced generic drugs. Because of their lower cost, the use of generic drugs is supported by health care systems which recommend that physicians prescribe them, rather than brand-name drugs [6–8]. Therefore, treatment of hypertension with generics is an ideal option to reduce health care costs and improve therapeutic compliance in Ethiopia.

### Lack of accredited bioequivalence center in Ethiopia

In most places in the world, an application for marketing approval of a new generic product must reference a corresponding product, which was approved on the basis of clinical trials to support claims of safety and efficacy. This means that generics must show bioequivalence (BE) to a reference product and this is accepted by the European Union (EU) [9], the United States of America (USA) [10] and the WHO [11]. However, due to the lack of a bioequivalence testing center in Ethiopia, whether locally produced drugs are therapeutically equivalent, and thus interchangeable with the originally marketed products, is not known.

One practical example for this, occurred when the Ethiopian medicines authority announced that the production of one particular locally manufactured drug was banned when it received claims of ineffectiveness of the drug from various health professionals [12]. Several studies have also reported high levels of negative perceptions regarding generic drugs among health professionals and patients in Ethiopia [13, 14].

### Enalapril

Enalapril is an angiotensin-converting enzyme (ACE) inhibitor used in the treatment of hypertension, symptomatic heart failure and asymptomatic left ventricular dysfunction [15]. Onset of antihypertensive activity is at 1 h, with peak reduction of blood pressure (BP) achieved by 4–6 h after administration. The extent of absorption of enalapril from enalapril tablets administered per os is approximately 60%. The effective half-life for accumulation of enalapril following multiple doses of the drug is 11 h. It is metabolized in the liver and excretion is primarily renal [16]. One study demonstrated that the first dose of enalapril was effective and produced effects similar to those measured after 7 days, and after 1 month of treatment [17]. In Ethiopia, enalapril is commonly used to treat hypertension and is one of the first-line treatment options in the routine management of patients with hypertension [18].

### What are N-of-1 trials?

N-of-1 tests are double-blinded, multiple-crossover, comparative trials of effect. They are indicated whenever there is substantial uncertainty regarding the comparative effectiveness of different treatments being considered for an individual patient. Guidelines commissioned by the US Department of Health and Human Services, and a recently published book have both documented the use of N-of-1 tests as a means of formally assessing the bioequivalence of generic drugs [19, 20]. Researchers have used N-of-1 tests to prove therapeutic interchangeability of generic warfarin [21] and nifedipine [22]. An N-of-1 trial also concluded that follow-up of hypertension using home BP measurement is possible [23].

N-of-1 trials are suitable in the following conditions: stable or chronic conditions; treatments that have a rapid onset and offset of effect, and a short half-life resulting in minimal washout periods [24, 25].. Enalapril meets all the conditions of a treatment that is amenable to N-of-1 trials [23].

### Justification for the trial

High-quality clinical evidence is required to prove the comparative efficacy and safety of locally produced enalapril compared with an accepted comparator, without a bioequivalence profile being present. Though they are cheap, people distrust local drugs. Pharmacodynamic (PD) endpoint bioequivalence studies can be used to establish bioequivalence of drug products when pharmacokinetic (PK) data is not possible [26, 27]. Compared with clinical endpoint bioequivalence studies, PD studies are more cost-effective, sensitive and less complicated methods for detecting drug formulation differences. Conducting classical clinical trials is difficult in Ethiopia. N-of-1 trials are relatively easy to conduct and generate results for each person. N-of-1 trials are not common in Africa. In particular, there are no published reports of N-of-1 methodology being used to assess the therapeutic equivalence (TE) of a product without a bioequivalence profile against another medicine that has a bioequivalence profile. If the tests prove feasible and acceptable, they can provide a bridge between the less rigorous regulatory environment in many resource-poor countries, and one in the future where bioequivalence tests are the norm.

N-of-1 tests prove TE and interchangeability in individual patients. Furthermore, when N-of-1 tests from multiple individuals are aggregated (aggregated N-of-1 tests), a smaller sample size is required to produce generalizable data compared to parallel randomized controlled trials (RCTs) [22, 28]. Therefore, as a proxy measure to a bioequivalence study, the aggregation of a modest number of patients who are undergoing N-of-1 tests could be used to determine overall TE in the population. To inform the design of this study, we conducted a preliminary qualitative study to assess the views of patients and physicians on local drugs. (publication in process) We identified that participants preferred imported drugs from Western countries, particularly Germany. For this reason, the German version of enalapril has been chosen as a comparator drug for this trial.

### Objectives

The objectives of the trial are to:

1. Compare the TE of locally produced enalapril and the German version of enalapril using N-of-1 TE tests (both versions are registered to treat hypertension in Ethiopia)
2. Assess whether N-of-1 TE tests are feasible to generate bioequivalence information for generic drugs in resource-poor settings

## Methods

### Trial design

This is a TE study using N-of-1 trials or tests. Each N-of-1 test will be a randomized, partially blind, multiple-crossover study designed to simulate the routine clinical practice of switching a patient between generic and brand forms of enalapril.

**Fig. 1:**
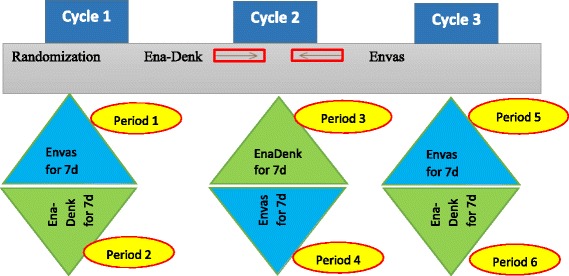
Diagram of N-of-1 test for treatment of hypertension using two enalapril formulations

**Fig. 2:**
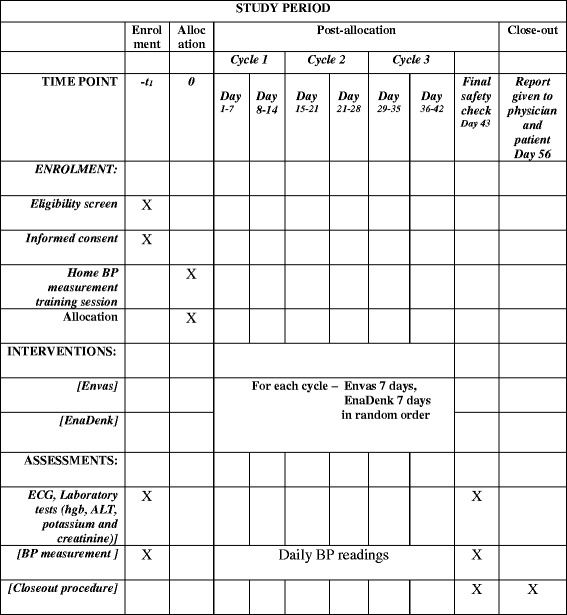
Standard Protocol Items: Recommendations for Interventional Trials (SPIRIT) schedule of enrollment, interventions and assessments

Each N-of-1 trial will consist of three successive 14-day treatment pairs, each pair comprising 7 days of Envas and 7 days of Ena-Denk in random order, taken once daily in the morning. The order of treatments in each treatment pair will be determined by block randomization. Figure 1 shows the study design for a sample arrangement of treatment order. The protocol conforms to the Standard Protocol Items: Recommendations for Interventional Trials (SPIRIT) guidelines [29]. The SPIRIT Checklist can be found in Additional file 1. Figure 2 shows the SPIRIT schedule of enrollment, interventions and assessments [29].

Blood pressure will be assessed morning and evening during the period, and by a study clinician at the end of each 7-day period. Blood pressure measurements taken in the first 2 days of each period will be discarded during the analysis stage to allow for washout.

### Study setting

This is a single-site, hospital-based study that will be conducted in the AHRI/ALERT center, one of the government-owned institutes located in the capital city of Ethiopia (Addis Ababa). The complex comprises the All Africa Leprosy, Tuberculosis and Rehabilitation Training Center (ALERT) specialized hospital and the Armauer Hanson Research Institute (AHRI).

### Study participants

Participants who fulfill the following inclusion criteria: (1) male and female patients with primary hypertension controlled on enalapril alone or an enalapril-containing regimen, and who have achieved a BP target of 140/90 mmHg or less in at least the last 2 months (clinic readings), (2) the enalapril dose is between 5 mg and 20 mg daily, (3) aged between 18 and 80 years, (4) serum electrolyte and creatinine within the normal range (or the clinical investigator considers the deviation to be irrelevant for the purpose of the study) and (5) a normal electrocardiogram (ECG) or stable abnormalities which the clinical investigator does not consider a disqualification for participation in the study.

Exclusion criteria include: (1) any evidence of clinically significant, poorly controlled hematologic, renal, hepatic, gastrointestinal, or diabetic problems, (2) any evidence within the last 6 months of clinically significant diseases involving the cerebrovascular, autoimmune, or cardiovascular systems, including poorly controlled angina pectoris, secondary hypertension, congestive heart failure, myocardial infarction or stroke, (3) concomitant use of major psychotropic agents or antidepressant drugs or regular use of non-steroidal anti-inflammatory agents, high-dose aspirin, or any agent that could raise or lower BP within the last 2 months, (4) a history of drug or alcohol abuse, (5) sensitivity to ACE inhibitors and (6) pregnant (positive pregnancy test) or breast-feeding women or women who are planning to become pregnant during the trial period.

## Interventions

### Investigational products

This N-of-1 TE study will compare two formulations of enalapril (Envas and Ena-Denk); see Table 1.

**Table 1:** Identity of investigational products

Description	Test product	Reference product
Name	Envas	Ena-Denk
Drug substance	Enalapril	Enalapril
Administration	Per oral	Per oral
Formulation	Tablet	Tablet
Dose	5–20 mg	5–20 mg
Manufactured by	Cadila Pharmaceuticals Ltd., Ethiopia	Denk Pharma, German

### Enalapril

For each patient, individualized enalapril will be prescribed for the duration of the study (6 weeks). Drugs will be bought from a well-regulated wholesale pharmacy. Study medications will be packed and labeled by an independent pharmacy unit in six opaque bottles containing 1 week’s supply of medicines each, and randomly ordered.

### Randomization

**Fig. 3:**
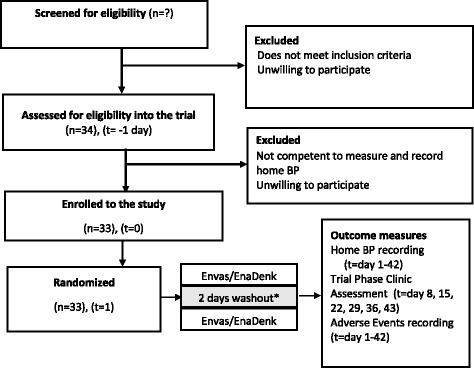
Flow of participants through the study. * This washout period is an analytic washout period. Blood pressure measurements taken in the first 2 days of each period will be discarded during the analysis stage to allow for washout

We will use block randomization, and with a block size of 2, by a statistician at The University of Queensland (UQ). The order in which patients receive drugs will be randomized by computer for each single case, such as BA-AB-BA or AB-BA-BA. The randomization schedule will be sent to a pharmacist who is independent of the study at the ALERT hospital. Prepared study medications will be delivered, stored and dispensed at the AHRI pharmacy unit. Randomization codes will be kept by the ALERT pharmacy. The codes will be broken by the Data Safety and Monitoring Board (DSMB) if safety concerns arise. Figure 3 shows the flow of participants through the trial.

### Blinding

Due to lack of encapsulation machines or matching placebo tablets locally, this N-of-1 study is planned to be a partially blinded trial. Patients may identify the physical differences between the two drugs, but will not know the identity of the medicines that they are taking unless the enalapril that they are currently prescribed is one of the test agents. This is possible but unlikely. However, the trial staff will remain blinded as study medications are prepared by a pharmacist who is independent of the trial. The importance of maintaining blinding is emphasized in the patient education that is a routine part of patient preparation for participation in the trial.

### Selection of doses in the study

Participants in this study are those whose BP is currently controlled with a regimen including enalapril 5–20 mg. They will continue on their current dose for the duration of the trial.

### Dose adjustment plan for uncontrolled BP

Standard clinic BP measurement is made at the end of each 1-week treatment period. Patients included in the study are those whose BP is controlled at the current dose and form of enalapril which they are taking. If a patient’s BP becomes less well-controlled, the dose of enalapril will be adjusted according to a preplanned protocol (see Table 2).

**Table 2:** Dose adjustment plan

Change in average BP	Initial dose	Dose adjustment	Maximum
< + 5 mmHg	Current dose	No adjustment – complete the cycle	20 mg
≥ + 5 mmHg	Current dose	Current dose + 5 mg	25 mg
≤ 5 mmHg fall	Current dose	No adjustment – complete the cycle	20 mg
≥ 5 mmHg fall and asymptomatic	Current dose	No adjustment – complete the cycle	20 mg
≥ − 5 mmHg and symptomatic	Current dose	Current dose − 5 mg	15 mg

*BP* blood pressure

### Concomitant medications

No new medications that could affect BP will be introduced for the duration of this study. Subjects should not take any traditional medicine for at least 2 weeks prior to commencing the study, and throughout the conduct of the study. During the study, subjects are advised not to take any other antihypertensive medications other than medications(s) that they were taking at the start of the study. If concomitant medication is unavoidable in case of emergency, the use must be reported in the patient’s diary (comment log) (dose and time of administration) and possible effects on the study outcome must be addressed (see also “Study withdrawal or interruption”). All other medicines that the patient takes will remain constant for the duration of the equivalence test.

### Education and instruction for subjects

To be included, participants should be able to demonstrate that they can measure and record their BP appropriately. Before randomization, each eligible participant will be trained using a standardized protocol for home BP measurement. Subjects will be trained about the basic methods of BP self-measurement, the meaning of BP values, and the monitoring device to be used by the principal investigator and study physician. Each patient will be given a digital home BP monitor. Subjects will be instructed to measure their BP three consecutive times in the sitting position in the morning before breakfast and in the evening after dinner. They will also be asked to visit the study site for follow-up on a weekly basis. They will be asked to take the respective study medication in the sitting position together with 250 ml water at the same time each day.

### Sample size estimation

The sample size for the individual N-of-1 trial is 30 BP readings (twice a day, three times each time) taken from the last 5 days of each trial period. The selection of a 5-day period of usable data in our study, and the number of BP readings required to sufficiently determine whether the local enalapril is therapeutically equivalent or not, is based on a previous N-of-1 trial of enalapril by Chatellier et al. [23]. Their investigation included two successive studies. In study 1, the variance components of BP and the choice of treatment period duration were determined in 35 hypertensive patients who remained untreated during the measurements. In study 2, a series of individual N-of-1 trials of identical design was performed after study 1 completion in 44 other consecutive patients. The first phase of the study reported that a period of five consecutive days of BP measurement is sufficient to accurately detect a drug-induced fall in BP in a single patient, provided that there are at least 30 readings in each 5-day trial period. However, the study did not consider the washout period and the individual agreement between the two cycles was only moderate at best. The authors recommended a trial of longer periods with three cycles. The 5-day period is a compromise between acceptability of the length of the individual trial and the reduction of variance allowed by the repetition of measurements with time.

An audit of sample sizes for pilot and feasibility trials undertaken in the United Kingdom reported that the median sample size for pilot trials was 30 participants per arm [30]. We assume that a sample size of 30 patients is adequate to assess feasibility. Considering a 10% dropout, a total of 33 participants will be included in the study. Only 10% dropout was considered based on the low dropout rate history from a previous cholera trial [31].

### Outcome measures

The study physician will assess the patient before and after each treatment period (each week) and collect home BP measurement and Side-effect Recording Forms from the previous week.

### Primary outcome

Therapeutic equivalence will be assessed by calculating change in mean seated systolic blood pressure (SBP). The minimal clinically important difference (MCID) is the smallest treatment effect that would lead to a change in a patient’s management [32]. Based on the academic literature [33], an average SBP difference of 5 mmHg is accepted as the MCID. A difference of < 5 mmHg in SBP between the two treatments is, therefore, considered to be of no clinical importance. Clinically important differences can be defined using a predefined MCID and confidence interval of the difference of the two treatments [34].

**Fig. 4:**
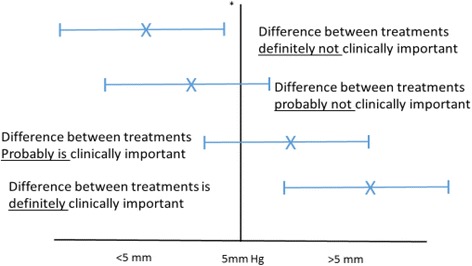
Mean clinical difference between two treatments (mean systolic blood pressure (BP)).  point estimate of the difference between groups,  95% confidence intervals, *minimum clinically important difference (MCID)

Clinically important differences in mean BP readings between the two treatments can have four interpretations, depending on the relationship of the MCID of the intervention to the point estimate (the best single value of the efficacy of the intervention that has been derived from the study results) and the 95% CI surrounding it [34] (see Fig. 4):

1. Definite – when the MCID is smaller than the lower limit of the 95% CI
2. Probable – when the MCID is greater than the lower limit of the 95% CI, but smaller than the point estimate of the efficacy of the intervention
3. Possible – when the MCID is less than the upper limit of the 95% CI, but greater than the point estimate of the efficacy of the intervention, and
4. Definitely not – when the MCID is greater than the upper limit of the 95% CI

### Criteria to establish therapeutic equivalence

1. Absence of a clinically important difference – for therapies to be considered equivalent, not only should the comparison of the efficacies of the two interventions not reach statistical significance, but also the upper limit of the confidence interval should be smaller than the predetermined MCID
2. Equivalence should be evident in at least in two cycles out of three

### Secondary outcomes

1. Change in mean home diastolic blood pressure (DBP) for both evening and morning diastolic BP values
2. Change in mean clinic DBP
3. Change in mean clinic SBP
4. Change in mean home DBP measured in the morning, 24 h after drug intake
5. Change in mean home DBP measured in the evening, 12 h after drug intake
6. Change in mean home SBP measured in the evening, 12 h after drug intake
7. Change in mean home SBP measured in the morning, 24 h after drug intake
8. *Feasibility outcome*: based on the academic literature [35], the following criteria will be used to measure success/acceptability of the pilot study:
  - Recruitment rate: at least 70% of all eligible patients can be recruited
  - Completion rate: at least 80% of all recruited subjects complete the study
  - At least 90% of patients took every scheduled dose of the study drug, and
  - More than 80% of requested measurements are obtained and valid. Measurements are considered invalid when either or both of systolic and diastolic BP readings are not compatible (less than 20 mmHg difference)
  - Patients’ and physicians’ views on the trial
9. *Safety outcomes*: adverse events (AEs) (number, severity) identified by patients recording. AEs recorded by the physician on the AE Case Report Form (CRF)

### Establishing controlled BP

In this study, those with an average clinic BP measurement controlled at 140/90 mmHg or less for up to 12 months will be included. This is because there is a considerable placebo effect for antihypertensive medicines, which increases after 1 year [36]. Hypertensive patients who have their follow-up in the ALERT hospital usually have a monthly visit schedule. To account for BP variations, including seasonal ones, BP will be established by taking the average of three consecutive BP measurements taken in at least the last 2 months.

### Using home BP measurement

BP self-measurement at home has been shown to be a sensitive tool, able to detect small changes, frequently resulting in reduced variability and greater reproducibility of measurements compared to office BP readings [37, 38]. Blood pressure self-measurement eliminates the white-coat effect [39]. It also provides better prediction of organ damage than clinic BP measurement [40, 41] and reduces the number of subjects necessary for meaningful results in drug trials [42, 43]. The average systolic BP over 5 days is used to assess the effectiveness of treatment. Frequent measurement of BP over 5 days narrows the variability of BP around the true mean BP value [44].^.^ Frequent measurement of BP improves diagnostic and management capacity [45, 46]. Blood pressure should be recorded immediately in a study diary.

### BP self-monitoring device

A clinically validated digital BP machine will be used for home blood measurement. A home BP measurement protocol is developed using standard methodology [47]. Before use by patients, the accuracy of each machine will be verified at the clinic/trial center.

### Safety evaluation and monitoring

Because this is a study of medication that the patient has been taking, the risk of serious adverse events is minimal. All abnormal or unpleasant effects from medications will be considered as side effects. Individual patients will collect common side effects using a questionnaire at home and bring it during their weekly visits. If a patient develops a serious side effect, the study physician will take appropriate measures. The most common side effects of enalapril include increased serum creatinine (20%), dizziness (2–8%), low BP (1–7%), syncope (2%) and dry cough (1–2%). The most serious common AE is angioedema (swelling) (0.68%) which often affects the face and lips, endangering the patient’s airway.

The DSMB consists of two senior members with clinical and methodological expertise who are independent of the trial. They will review all documented harms during the study and adjudicate them with regard to causality.

### Study withdrawal or interruption

If a concomitant medication has to be taken that might influence BP, that person will cease the trial until the new medicine has ceased or the dose has been stable for at least 2 weeks. If they wish, the participant can restart the study, substituting a new randomly packed cycle of medicines for the interrupted cycle. Unused medicines from the trial will be discarded. If the person has to continue on the new medicine, the trial will cease, and the data collected will be analyzed if at least one cycle has been completed.

Participants will also be withdrawn from the study under the following conditions:

- Patient request, patient non-compliance, or the development of an exclusion criterion
- If, in the opinion of the treating clinician, the patient’s interests are best served by withdrawing from the trial

### Data analysis and study result report

#### Individual study result report

At the end of the trial, BP measurements will be analyzed and the result will be given to the treating physician. After looking at these results, the patient and the treating physician will be able to decide if the local drug works for treating the patient’s hypertension.

#### Statistical methods

Analysis will be conducted based on BP measurements taken in the last 5 days of each of the six periods. The first 2 days’ BP measurements in each period will be discarded. Based on the academic literature [33], 6 mmHg is accepted as the MCID. Therefore, a SBP difference of ≥ 5 is considered clinically significant. A mean change in evening SBP of < 5 mmHg between the two test medicines in at least two treatment cycles will be considered as representing TE.

Differences in treatment means will be explored graphically, and through the paired Student’s *t* test as appropriate. Significance will be set at *P* < 0.05. The data will also be modeled within a linear mixed-effects modeling framework to account for repeated measures over time such that between- and within-patient variability can be captured (and estimated). If the modeling provides evidence that sources of variability are significant (e.g., possible relationships between the three readings at each morning and evening), then appropriate adjustments built in to the model will be retained. From such a model, population and individual estimates of treatment means can be obtained, and the influence of additional variables. such as treatment order. can be accounted for (if needed). This statistical analysis will be undertaken in the R package.

The type and severity of possible side effects of the study medication and any serious adverse events will be tabulated for individual patients.

#### Data management

Data that will be generated in this study will be appropriately documented and checked for validity and accuracy. Study data will be managed using the R package. Data from paper CRFs will be double entered into the database by two persons independently so that data will be matched and checked for validity and accuracy before being endorsed for analysis. Problems with incomplete and missing data will be resolved in the following ways. If a patient missed a day of BP measurement, we will provide a mean score for that period. However, if there are more than or equal to 2 days’ data missing, we will not provide a score for that period and that will be indicated in the patient’s report. As per International Conference on Harmonization (ICH) Good Clinical Practice (GCP) guidelines [48], the investigators will maintain information in the study subjects’ records which corroborates the data collected and entered into the CRFs.

## Discussion

The numbers of local pharmaceutical industries that market their products, such as enalapril, without proof of bioequivalence are dramatically increasing. The Ethiopian guideline for registration of medicines states the need for clinical trial evidence on occasions when PK bioequivalence is not applicable. The guideline states that when in-vivo studies using plasma concentration time-profile data are not suitable, a comparative clinical trial then has to be performed to demonstrate equivalence between two formulations. However, as stated in the background, in East Africa including Ethiopia, local generics are marketed without evidence of effectiveness obtained through bioequivalence studies or clinical trials.

By bridging the information gap on bioequivalence, the trial will give rigorous evidence on TE of local enalapril in individual patients. If there is no difference between the two treatments (the hypothesized result), then patients can take the local cheaper medicine with confidence. This trial will also will determine whether aggregated N-of-1 studies are feasible to provide a population level estimate of clinical equivalence for untested generic drugs in resource-limited countries where bioequivalence testing centers are unavailable. If TE is shown, it will encourage the use of cheaper local drugs by enhancing reliance on local pharmaceutical companies.

However, there are a number of challenges in the design and conduct of reliable N-of-1 TE studies. Generally, comparison of drug formulations using TE studies are recommended if the PK approach is not possible [26, 27]. TE studies demonstrate that a test formulation has equivalent efficacy to a reference one. Often times, TE tests using clinical endpoints are less reliable due to lack of clearly defined endpoints and huge variability in the measured parameters [26]. Therapeutic equivalence studies with biomarkers reflecting efficacy endpoints (e.g., BP reduction) are more sensitive than TE studies with clinical endpoints (e.g. survival rates, myocardial infarction and stroke) [26, 27]. Beside, N-of-1 trials are suitable only in chronic conditions where the drugs or interventions that are to be tested have a relatively short half-life [24, 25]. The use of technology (e.g., mobiles, BP machines, etc.) has a significant contribution in standardizing treatment measures. We are comparing antihypertensive effect (maintenance of BP) which is amenable to the use of these reliable technologies.

Patients involved in this trial are not blinded. While double blinding is an ideal option, the need to adapt to local conditions is also very important. Apart from logistic issues (lack of an encapsulation machine locally), keeping the design less complex would make the trial resemble the usual way of switching drugs except that it is pre-planned, randomized and closely monitored [49]. Our method of unblinding of patients and keeping study clinicians and investigators blinded is a compromise between feasibility and minimal potential effects on reported outcomes. The outcome of the study (BP) could be affected more than other possible, but not feasible, outcomes (e.g., serum renin levels). On the other hand, the use of electronic BP devices will help in standardizing the measure. The importance of maintaining blinding is part of patient education that is a routine part of patient preparation for participation in the trial. Study medications are prepared by an independent pharmacy unit. Therefore, study investigators and DSMB members will be blinded. A review of the relevant academic literature found that three N-of-1 papers, including one protocol publication on hypertension, were not fully blinded [22, 23, 49].

## Trial status

Currently, 33 patients are enrolled and we expect the study to be completed by 7 October 2017.

### Sponsor

The trial sponsor is The University of Queensland. The sponsor has no role in the trial design; collection, management, analysis or interpretation of data; writing of reports; or submission for publication.

## Additional files

Additional file 1: SPIRIT 2013 Checklist: recommended items to address in a clinical trial protocol and related documents. (DOCX 50 kb)

Additional file 2: Patient Information Sheet and Informed Consent Form. (DOCX 99 kb)

## Abbreviations

AE: Adverse event
AAERC: AHRI/ALERT Ethics Review Committee
ACE: Angiotensin-converting enzyme
AHRI: Armauer Hanson Research Institute
BE: Bioequivalence
CI: Confidence interval
CRF: Case Report Form
DBP: Diastolic blood pressure
ECG: Electrocardiography
ERC: Ethics Review Committee
EU: European Union
FMOH: Federal Ministry of Health
ICH: International Conference on Harmonization
IRB: Institutional Review Boards
MCID: Minimal clinically important difference
PD: Pharmacodynamic
RCTs: Randomized controlled trials
SPIRIT: Standard Protocol Items: Recommendations for Interventional Trials
SBP: Systolic blood pressure
TE: Therapeutic equivalence
UQ: University of Queensland
USA: United States of America
WHO: World Health Organization
