# Supplementary material for: A series of N-of-1 trials to assess the therapeutic interchangeability of two enalapril formulations in the treatment of hypertension in Addis Ababa, Ethiopia: study protocol for a randomized controlled trial
Source: Trials. 2017 Oct 10;18:470. doi: 10.1186/s13063-017-2212-0 (PMC5634952; doi:10.1186/s13063-017-2212-0)
Supplement: Supplementary file 2 — Patient Information Sheet and Informed Consent Form. (DOCX 99 kb) [file 13063_2017_2212_MOESM2_ESM.docx]

#

# **PARTICIPANT INFORMATION SHEET**

**STUDY TITLE:** A series of N-of-1 trials to assess therapeutic interchangeability of two Enalapril formulations in the treatment of hypertension in Addis Ababa, Ethiopia: study protocol for a randomised controlled trial

**List of investigators:**

| **Name** | **Affiliation** | **Email** | **Contact** |
| --- | --- | --- | --- |
| Mr Chalachew Alemayhu (Chief investigator) | School of Medicine University of Queensland | c.alemayehu@ uq.edu.au | Tel: +251-911535973 |
| Prof Geoff Mitchell | School of Medicine University of Queensland | g.mitchell@uq.edu.au | Tel: 07 33811363 |
| Dr. Abraham Aseffa | Armauer Hanson Research institute | [aseffaa@gmail.com](mailto:aseffaa@gmail.com) | Tel: (251)-113483752 |
| Associate Professor Alexandra Clavarino | School of Pharmacy University of Queensland | [a.clavarino@pharmacy.uq.edu.au](mailto:a.clavarino@pharmacy.uq.edu.au) | Tel:  7-334-61979 |
| Dr James McGree | Queensland Univerity of Technology | [james.mcgree@qut.edu.au](mailto:james.mcgree@qut.edu.au) | Tel: 07 3138 2313 |
| Dr Jane Nikles | UQCCR | uqjnikle@uq.edu.au | Tel: 073346 5025 |

- **Invitation**

We are inviting you to participate in N-of-1 tests to assess whether two Enalapril formulations work equally well in the treatment of hypertension in Ethiopia. These tests are useful to see whether the local product works as well as the imported one and if so, it could be taken confidently by individual patients like you.

The study is being conducted by Mr Chalachew Alemayehu, a PhD student at The University of Queensland, Australia, in collaboration with The Armauer Hansen Research Institute (AHRI) in Ethiopia.

**Project overview**

Many patients with hypertension take Enalapril to control their blood pressure. Using locally produced Enalapril is a cheaper option for patients with hypertension. However, there is no evidence that proves that locally produced Enalapril works as well as the original (standard) Enalapril. The aim of the trial is to test whether locally produced Enalapril works as well as imported Enalapril in the treatment of hypertension.

**What is Enalapril?**

Enalapril is the preferred drug for the management of hypertension and a commonly prescribed drug for the treatment of hypertension in Ethiopia. You may be taking this medication either singly or combined with other anti-hypertensive drugs/s.

**What is Nof-1 test?**

N-of-1 tests are a means of assessing whether a medication works for the patient who takes the medicine. N-of-1 tests systematically assess the relative effectiveness of various treatment options in an individual patient so as to identify the best treatment option. If two treatment options work as well as each other, the less costly drug can be chosen.

The N-of-1 test will take six weeks and will use locally produced Enalapril and imported Enalapril alternatively on a weekly basis.

**Why the Nof-1 test is needed?**

Currently, CADILA Pharmaceuticals Ltd (Ethiopia) produce Enalapril (Envas) in Ethiopia. However, to ensure its effectiveness, the comparative efficacy and safety of this drug againist the original (standard) product should be tested.. Currently, appropriate tests to prove effectiveness are not available in Ethiopia.

**What happens if I decide to participate?**

**Before trial commencement:**

***Screening phase***

We want the participants in this study to meet a certain level of health. Therefore, a screening examination will be done two days before the start of the study. We will do a physical examination to check your general health condition. We will take one blood sample (10 ml/2 tsp) from your arm, and we also want you to take one urine sample (15 ml/3 tsp). We will then analyse the blood and urine samples with standard clinical tests to assess your liver and kidney functions and blood values.

An electrocardiogram will be also performed to assess whether there are significant cardiac abnormalities. If you are a female, we will also perform a pregnancy test. If you are pregnant or plan to become pregnant during the next 2 months you cannot participate in this study.

If you can’t read and write, you should have a willing assistant that can help you manage BP monitoring and data collection forms throughout the course the study.

***Final eligibility assessment***

If you pass the above screening phase, a BP training session will be conducted one day before the start of the study. The training is about the basic methods of BP self-measurement, the meaning of BP values, and the monitoring device to be used. Your or your assistant’s competence and skill will be assessed after the training. Then if you are eligible, you will be included.

**During the trial phase**

Seven tablets of Enalapril (either test product or the reference product) will be dispensed to you each week for six weeks. You will be taking each medication in **random (mixed up) order.** .

**Example arrangement of treatment order**

| Week 1 | Week 2 | Week 3 | Week 4 | Week 5 | Week 6 |
| --- | --- | --- | --- | --- | --- |
| Treatment  1 | Treatment  2 | Treatment  3 | Treatment 4 | Treatment  5 | Treatment  6 |
| 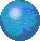 | 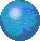 | 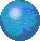 | 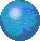 | 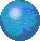 | 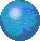 |
| Ena-Denk | Envas | Envas | Ena-Denk | Ena-Denk | Envas |

You will be given a home BP monitoring device and recording form. You will measure your BP three consecutive times in the sitting position in the morning before breakfast and in the evening after dinner. You will measure your BP at home for successive six weeks. You will record your BP immediately after each measurement in a study diary. The average systolic (top) and diastolic (bottom) BP at home over five days will be used to assess the effectiveness of treatment. You will bring your BP recording diary during your weekly clinic visit.

You will abstain from taking any new medication (prescription and nonprescription drugs) without notifying the study staff. If you smoke and/or take alcohol, you need to minimize your consumption.

You will visit the study site for follow up on a weekly basis. During these visits, you are expected to bring recordings which are made during the last week. During the trial phase, you will visit the clinic six times.

- Visit 1: At the end of the first week (Day 7)
- Visit 2: At the end of the second week (Day 14)
- Visit 3: At the end of the third week (Day 21)
- Visit 4: At the end of the fourth week (Day 28)
- Visit 5: At the end of the fifth week (Day 35)
- Visit 6: At the end of the sixth week (Day 42)

**What would happen after the N of -1 trial?**

**At** the end of this trial, the treating physician will be told when you were taking the locally Enalapril (Envas) and the time you were taking the reference Enalapril. Your BP measurements will be analysed and the result will be given to the treating physician. After looking at these results, you and the treating physician will be able to decide if the local drug works as well as the reference medication for your hypertension.

You will be asked to fill a short questionnaire at the end of the study, to ask you about your experiences of being in the study. It should take no more than ten minutes.

**Potential risks**

Enalapril is the drug you have already been taking to control your BP. In general, it is one of the safe drugs to take. If you have not experienced side effects till now, it is most unlikely you will start to experience them in the trial.

But still, as with any drug, there are some side effects. The most common side effects of Enalapril include mildly reduced kidney function (20%), dizziness (2–8%), low blood pressure (1–7%), syncope (fainting) (2%), and dry cough (1–2%). The most serious common adverse event is swelling of the mouth and lips (0.68%), which can make breathing difficult. All unpleasant effects from medications will be considered as side effects. You are encouraged to record any unpleasant effects and take the record to your weekly clinic visit. A side effect questionnaire will be given to you.

**What I should do if I have any concerns about medication side effects?**

If you have any concerns about the medication please contact the chief investigator on the phone number at the beginning of this information sheet.

**Benefits to the participant:**

This trial will give you and the treating physician clear information as to whether there is any clinically important difference between the two forms of Enalapril. If not, then the treating physician will be able prescribe the less costly drug and you will be able to take it confidently. Moreover, during the course of the study, you and the treating physician will be able to frequently monitor your blood pressure - which is vital to be able to understand your condition well.

**Expenses and compensation**

You will be reimbursed for the direct expenses related to travel to the study site and loss of earnings during the visit days. The total amount of money to be compensated is 150 Eth Birr per visit.

**Confidentiality and data protection**

All personal records will be kept confidential and kept locked in cabinets. Your identity and other identifiable information will only be known to the clinical trial staff. Data from this project may be published or presented. No personal information will appear in any report from this study. All test results will be treated confidentially with use of coded labels on samples. A separate file with no identifying data will be made for analysis.

**What happens if I don’t want to take part in this study?**

Your participation in this study is absolutely voluntary. If you decide not to participate, it will not affect the treatment you receive now or in the future. If you decide to stop participating during the study, you can leave the study at any time without providing any reason.

**Consent**

A Consent Form must be signed.

**Ethics approval**

# Ethics approval for this project has been granted from The University of Queensland Human Research Ethics Committee (Australia). Additionally, this project has been approved in Ethiopia by AHRI/ALERT ethics committee.

If you would like to contact AAERC secretariat, use this number: +251-118-962183 or you can speak to an officer of the University not involved in the study, you may contact the Ethics Officer on +61 7 3365 3924 or you can email for the human ethics office: [humanethics@research.uq.edu.au](mailto:humanethics@research.uq.edu.au). )

This study has been supported by The University of Queensland

**Feedback to participants**

Once the last participant has finished the study treatment and all data from this research have been analysed, we will be able to discuss the overall findings with you the treating physician on request.

If you have any reservations about you and/or the treating physician being informed about this study and the results, please let us know of your concerns.

**You are encouraged to ask questions at any time during your participation in the study.** You are of course, free to discuss your participation in this study with Mr Chalachew Alemayehu on +251-911535973.

#

#

# **PARTICIPANT CONSENT FORM**

**STUDY TITLE:** A series of N-of-1 trials to assess therapeutic interchangeability of two Enalapril formulations in the treatment of hypertension in Addis Ababa, Ethiopia: study protocol for a randomised controlled trial

I confirm that:

- I have read, or have had read to me, and I understand the Participant Information and Consent Form;
- Had any questions or queries answered to my satisfaction;
- Been informed that one blood sample (10 ml/2 tsp) and one urine sample (15 ml/3 tsp) are required and an electrocardiogram will be performed during the screening phase;
- Been informed that there will be a BP training session one day before the start of the study;
- Been informed of the possible risks or side effects of the medications, tests or procedures being conducted;
- Understood that participating in this study will require me to take a tablet every day that will contain either the local Enalapril (Envas) or reference drug (Ena-Denk);
- Been informed that a total of 150 Eth Birr per visit will be reimbursed for my direct expenses related to travel to the study site and loss of earnings during the visit days;
- Been informed that, during the trial phase, a total of six visits are required to the study site;
- Been informed that I will measure my BP two times a day for consecutive six weeks at home;
- Been informed that the confidentiality of my information will be maintained and safeguarded; and
- Agreed to participate in the study.
- I am happy for the treating physician to be told of my results: Yes No
- It is explained to me that in case I can’t read and write there will be a witness (who does not belong to the investigators) whose role will be to read, understand and explain the study concept to me. To this end, it has been read to me before my participation to the study.
- It is explained to me that in case I can’t read and write I should have a willing assistant who can help me throughout the duration of the study.

I: _______________________________________________________freely choose to take part in this study.

(Name and surname of participant in capitals)

Participant's signature: ________________________________________________________

Date: _____/_____/______

I: ___________________________________________________ was present when the form was completed and confirms the participant has understood.

(Name and surname of witness in capitals)

Witness' signature: __________________________________________________________

Date: _____/_____/______

I: __________________________________________________________________

(Name and surname of the Principal Investigator or appointed person)

Have carefully explained the requirements, risks and benefits foreseen in the study to the aforementioned persons and I was present when this form was completed.

Signature of Principal Investigator (or appointed person):_______________________

Date: _____/_____/______

**A copy of the Information sheet and the signed Consent Form will be provided to the participant.**
